# Abnormal correlation between phase transformation and cooling rate for pure metals

**DOI:** 10.1038/srep22391

**Published:** 2016-03-04

**Authors:** J. J. Han, C. P. Wang, X. J. Liu, Y. Wang, Z.-K. Liu, T.-Y. Zhang, J. Z. Jiang

**Affiliations:** 1 Fujian Key Laboratory of Materials Genome, College of Materials, Xiamen University, Xiamen 361005, P. R. China; 2 Collaborative Innovation Center of Chemistry for Energy Materials, Xiamen University, Xiamen 361005, P. R. China; 3 Department of Materials Science and Engineering, Pennsylvania State University, University Park, Pennsylvania 16802, USA; 4 Shanghai University Materials Genome Institute and Shanghai Materials Genome Institute, Shanghai University, 99 Shangda Road, Shanghai 200444, China; 5 International Center for New-Structured Materials (ICNSM), Laboratory of New-Structured Materials, State Key Laboratory of Silicon Materials, and School of Materials Science and Engineering, Zhejiang University, Hangzhou, 310027, P. R. China

## Abstract

This work aims to achieve deep insight into the phenomenon of phase transformation upon rapid cooling in metal systems and reveal the physical meaning of scatter in the time taken to reach crystallization. The total number of pure metals considered in this work accounts for 14. Taking pure copper as an example, the correlation between phase selection of crystal or glass and cooling rate was investigated using molecular dynamic simulations. The obtained results demonstrate that there exists a cooling rate region of 6.3 × 10^11^–16.6 × 10^11^ K/s, in which crystalline fractions largely fluctuate along with cooling rates. Glass transformation in this cooling rate region is determined by atomic structure fluctuation, which is controlled by thermodynamic factors. According to the feature of bond-orientation order at different cooling rates, we propose two mechanisms of glass formation: (i) kinetic retardation of atom rearrangement or structural relaxation at a high cooling rate; and (ii) competition of icosahedral order against crystal order near the critical cooling rate.

Metallic glasses (MGs) are one of the most intensively studied advanced materials due to their scientific significance and potential engineering applications^1^. Most MGs are fabricated from the molten liquids on cooling by avoiding nucleation and growth of crystalline phases. Since metallic liquids have a strong tendency to crystallize when cooled below the liquidus temperature, MGs are thermodynamically metastable. Thus, the formation of MGs and its competition with crystallization have aroused considerable interests. When metallic liquids solidify at high cooling rates, nucleation can be suppressed, producing metallic glasses with an amorphous structure. Under such circumstances, the liquid structure with a short-range order remains in a solid amorphous state without long-range translation order. Most alloys require a critical cooling rate of about 10^6 ^K/s for the formation of an amorphous structure^1^,^2^,^3^, while pure-element metals require critical cooling rates as high as 10^12 ^K/s. In principle, the critical cooling rate for forming glass for a given system can be estimated from the position of the “nose” on the time-temperature-transformation (TTT) diagram^4^. It is widely believed that using a cooling rate higher than the critical one, any liquid can be turned into an amorphous solid. The glass transformation is thus regarded as a process in which crystallization is kinetically retarded within a certain time period. In this sense, metal liquid quenched by faster cooling rate could retain more random structure than that quenched by slower cooling rate, as the transient nucleation theory points out^5^,^6^.

Differing from the crystalline counterpart, whose configuration space is dominated by a unique free-energy minimum that corresponds to a perfectly ordered structure, the glass may take on a large number of equivalently disordered, imperfect structures^7^. This consequence is resulted from the thermal fluctuation, along with structural fluctuation, occurring in supercooled liquid upon cooling. In the framework of random first-order transition (RFOT)^8^, dynamics of supercooled liquid is associated with the transitions between amorphous free-energy minima, separated by respective barriers. It pointed out that the slowdown of dynamics as temperature decreases is caused by the fact that fewer configurations are available, and the kinetic pathways between them become more complex and more collective^8^. Supported by mean-field calculations^9^, the thermodynamic-kinetic correlation of liquid-to-glass transition was well established^10^,^11^. However, the cooling-rate dependence of such correlation is hardly involved in these theories, and also the picture of phase transformation (glass or crystal) at critical point of phase selection is unclear.

Recently, it has been pointed out that the stability of supercooled liquid against crystallization, which is associated with the structural heterogeneity level^12^, is affected by the liquid’s thermal history. Following this scenario, nucleation density is sensitive to the atomic structures in the as-prepared supercooled liquid^13^, which yields different pathways of crystallization. This could be actually related to the scatter of the onset time for nucleation in the process of isothermal annealing for metallic liquid systems, as reported by experimental measurements and theoretical predictions in literatures^14^,^15^. Therefore, it is reasonable to believe that structural fluctuation plays an important role in the glass transformation or crystal nucleation. The phase transformation of supercooled liquid would be determined by the coupling of structural fluctuation and arrest of dynamics. In views of the time-scale dependence of structure relaxation, this coupling is predicted to be significant particularly near the critical cooling rate for phase selection. Until now, the effect of the thermodynamic-kinetic correlations on the phase transformation within this region has not been well understood. How the coupling of structural fluctuation and arrest of dynamics determines the phase transformation in supercooled liquid is still open. To address this issue, in the present work, we choose pure-element metal systems, including Cu, Ag, Au, Ni, Pd, Pt, Al, Pb, Fe, Mo, Ta, W, Ti, and Zr, to investigate the effect of structural fluctuation on phase transformation at cooling rates near the critical point, so that the compositional fluctuation factor can be eliminated. We examined the structural origin and physical meaning of the scatter of onset time of nucleation, based on classical molecular dynamics (MD) simulation with the embedded atom method (EAM) as proposed by Mendelev^16^ and supplied in LAMMPS^17^. The atomic-scale mechanism underlying the phase transformation as a function of cooling rate is characterized by adaptive common neighbor analysis (a-CNA)^18^ and local bond-orientational order (BOO) parameters^19^. Due to the similar results and consistent conclusions from these pure-element metal systems, here we only present the results of copper.

## Results and Discussion

### Critical cooling rates from TTT diagram

**Figure 1:**
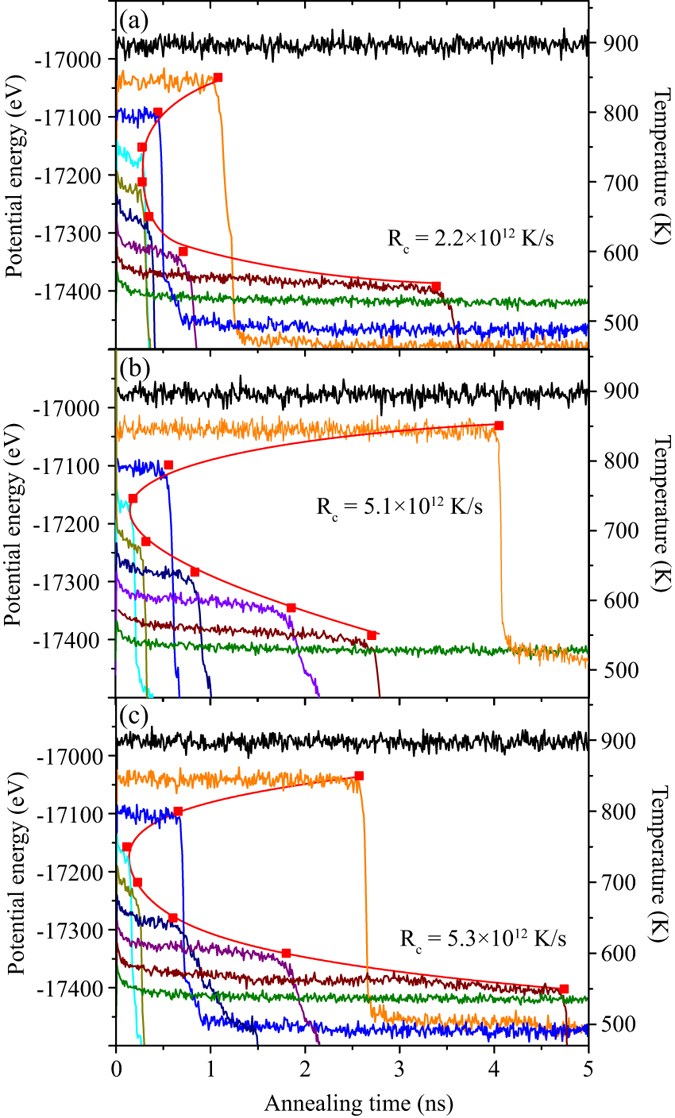
Time dependence of potential energy during annealing at different temperatures for three independent samples. The different color lines represent the potential energies at different temperatures. The red curve represents a typical TTT diagram, showing the relationship between the onset time of the potential energy drop and the temperature. The critical cooling rates *R*_c_ for the glass transitions are estimated to be (**a**) 2.2 × 10^12 ^K s^−1^, (**b**) 5.1 × 10^12 ^K s^−1^, and (**c**) 5.3 × 10^12 ^K s^−1^, respectively.

Figure 1 shows the relationship between time and potential energy during isothermal annealing at temperatures from 500 K to 900 K for three independent samples of supercooled liquid copper. Since the appearance of crystal nucleation accompanies with a sudden change of potential energy^20^, the deflection points of potential energy marked by red squares in Fig. 1 correspond to the times needed to begin the crystallization process at annealing temperatures. Figure 1 clearly illustrates that at a given temperature the onset times for crystallization differ from each other in the three independent samples of supercooled liquid copper. These results suggest that the initial structure in liquid exerts a significant influence on the nucleation process, in accord with the previous work^14^. Similar results were also observed in experiments using repeated isothermal methods and isolated particles in multi-component metal systems^4^,^15^. The TTT diagram shows the typical “nose” shape for the onset time of crystallization. The critical cooling rates *R*_c_ for the glass transformation are estimated to be 2.2 × 10^12^ K/s, 5.1 × 10^12 ^K/s, and 5.3 × 10^12 ^K/s, respectively, by applying isothermal annealing temperatures to the equation 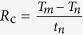, where *t*_n_ is the nose time, *T*_m_ the melting point, and *T*_n_ the nose temperature determined from the TTT diagram. Due to the non-linearity of nucleation and the existence of an induction time for nucleation, the values of *R*_c_ according to the nose method from the TTT curves are always larger than those determined from constant cooling rate conditions^21^. Therefore, the above estimated critical cooling rates for the vitrification of melted copper could be the up-boundary of the actual required cooling rate. Thus, simulations of continual cooling were accordingly performed at cooling rates ranging from 10^11 ^to 10^13 ^K/s.

### Abnormal correlation between phase selection and cooling rate

**Figure 2:**
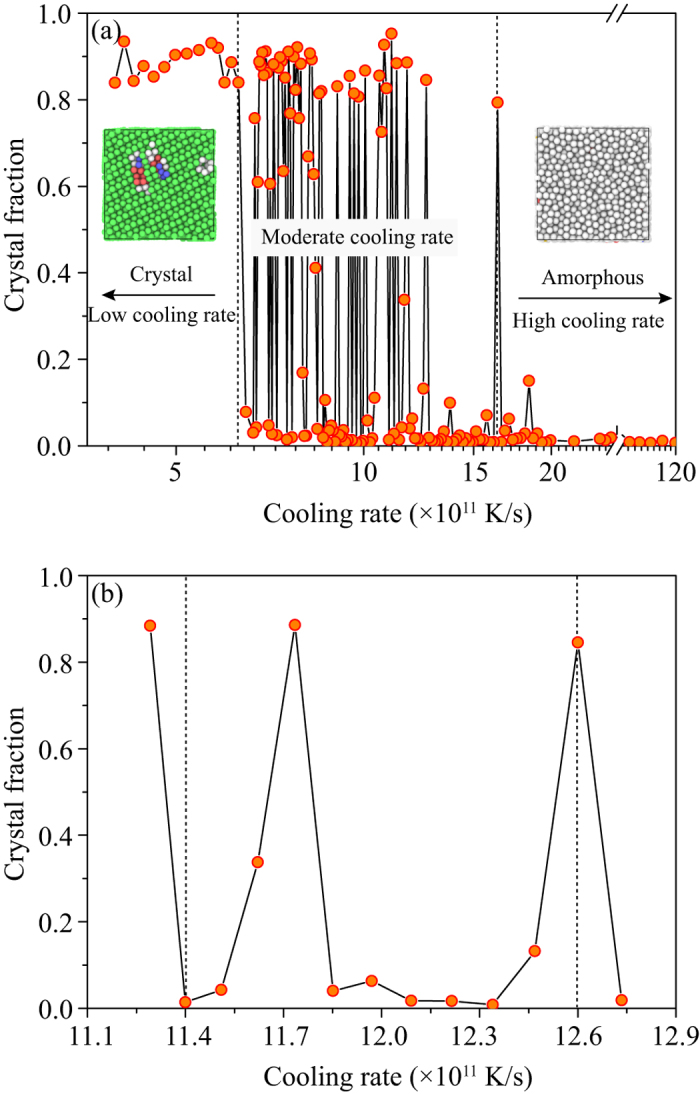
Crystallinity in samples as a function of cooling rates. (**a**) The cooling rates range from 119.7 × 10^11 ^K/s to 4.0 × 10^11 ^K/s, with (**b**) a magnified intervals from 11.3 × 10^11 ^K/s to 12.7 × 10^11 ^K/s for clarify. The two images in (**a**) are typical amorphous structure (right) and crystalline structure (left), characterized by a-CNA. Atoms with FCC-like environment are represented by green, HCP-like by red, BCC-like by blue, icosahedra-like by yellow and others by grey.

Figure 2 shows the fractions of crystalline component in the cooled Cu sample as a function of cooling rate ranging from 4.0 × 10^11 ^K/s to 119.7 × 10^11 ^K/s after cooling from 1495 K to 298 K. Atomic structures, e.g., face-centered cubic (FCC-like), hexagonal close-packed (HCP-like), and body-centered cubic (BCC-like) crystalline structures, as well as other amorphous structures, can be identified during the entire cooling process using a-CNA, as shown in the images included in Fig. 2a. It should be noted that since the atoms in grain boundaries might be identified as amorphous structures in the samples after rapid cooling, the crystalline fraction in cooled samples can scarcely reach 100% even if the liquid crystallizes completely. It is found that samples always contain a high crystalline fraction when cooling rates lower than 6.3 × 10^11 ^K/s were used. In this cooling rate region, the transient nucleation frequency appears to have little influence on structural evolution, since the rate of nucleation relaxation exceeds that of the change of nucleation barrier during cooling^6^. In contrast, nucleation relaxation through atom rearrangement is retarded by cooling rates higher than 16.6 × 10^11 ^K/s. In this cooling rate region, the high cooling rate prevents the liquid from crystallization, and glass transformation occurs. These observations are in accordance with the general kinetic perspective on phase transformation^5^. Surprisingly, we detect an abnormal cooling rate effect on phase transformation in a narrow cooling rate region between 16.6 × 10^11 ^K/s and 6.3 × 10^11 ^K/s, where crystalline fractions can largely fluctuate with the cooling rate. By viewing Fig. 2b, it is clear that the phase transformation is highly sensitive to the cooling rate, and the crystal fraction keeps fluctuating in this cooling rate region. More significantly, a faster cooling rate would no longer guarantee a lower crystal fraction than a slower cooling rate. For example, the crystal fraction of solid quenched by a cooling rate of 12.6 × 10^11 ^K/s is higher than that by a cooling rate of 11.4 × 10^11 ^K/s. To confirm these results, different simulation conditions including initial structures and system size for various pure metal systems have been carried out and it can be found that this phenomenon is universal (Supplementary Figs S1 and S2). This fact reveals that the general rule of thumb is broken within this cooling rate region, strongly suggesting that the phase transformation in this moderate cooling rate region is associated with the evolution of atomic structure, not controlled by the kinetic factor but the thermal history by different cooling rates.

### Atomic structure evolution upon rapid cooling

**Figure 3:**
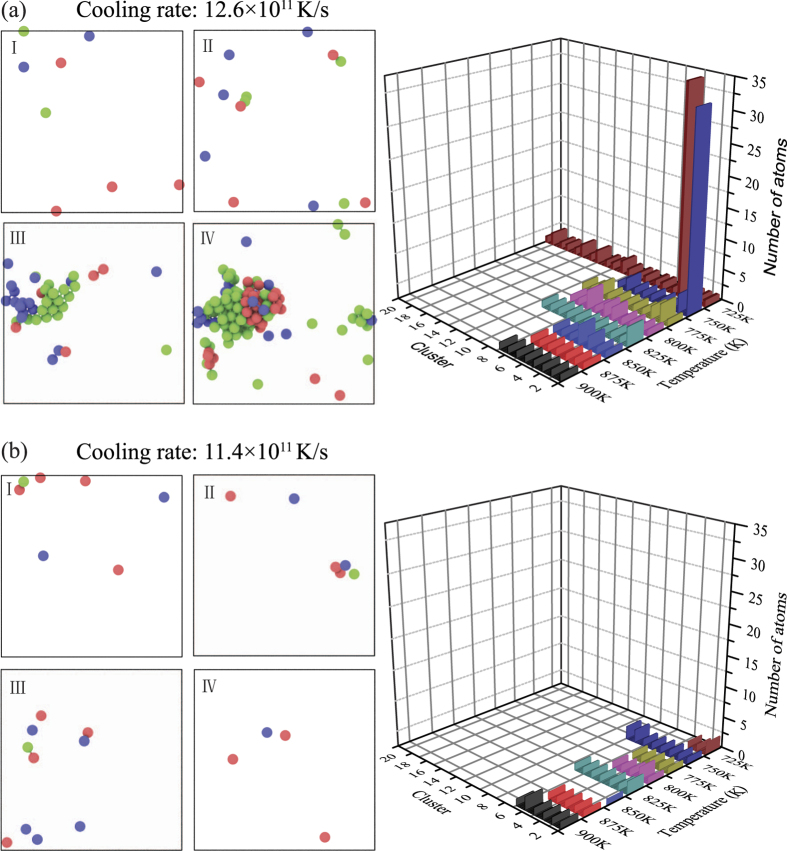
Structures and sizes of clusters as a function of temperature. The images from top to bottom and left to right on the left side represent temperatures at 900 K, 800 K, 750 K, and 700 K. (**a**) The size of clusters as a function of temperature for a cooling rate of 12.6 × 10^11 ^K/s; (**b**) the size of clusters as a function of temperature for a cooling rate of 11.4 × 10^11 ^K/s.

**Figure 4:**
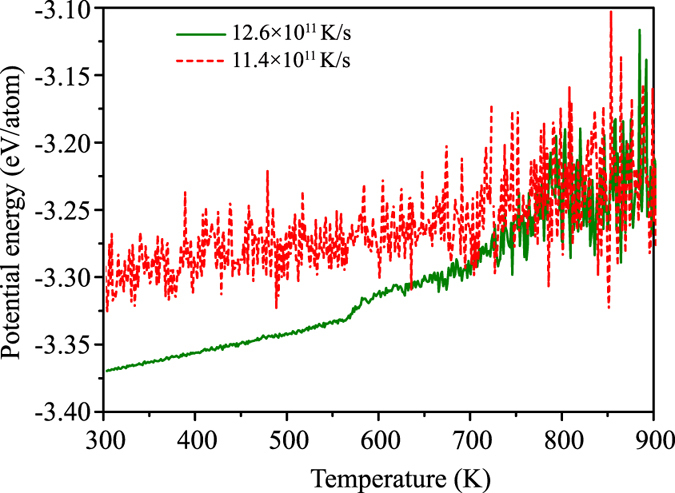
Average potential energies of atoms in crystal-like clusters. Average potential energies of atoms in crystal-like clusters as a function of temperature for both cooling rates of 12.6 × 10^11 ^K/s, and 11.4 × 10^11 ^K/s.

To visualize the cooling rate dependence of atomic structure evolution, Fig. 3 captures the distributions of crystal-like clusters at a faster cooling rate of 12.6 × 10^11 ^K/s and a slower cooling rate of 11.4 × 10^11 ^K/s. At the beginning, the isolated crystal-like clusters seem to emerge randomly from liquid at a comparable pace for both cooling rates. Some early-formed clusters dissolve as other ones emerge, no matter of which type of crystalline structure. When temperature is below 750 K, some stable crystal-like nuclei are formed. Surprisingly, the nuclei that reach the critical size actually appear at the faster cooling rate of 12.6 × 10^11 ^K/s, rather the slower rate of 11.4 × 10^11 ^K/s. This indicates that in this cooling rate region, nucleation and growth could be determined by thermal history in terms of structural fluctuation rather than by dynamics like diffusion for pure copper. Monitoring the nucleation process in terms of the size variation of crystal-like clusters could provide insights on the thermodynamics and kinetics of nucleation during a complex temperature-time history. The right side plots in Fig. 3 show the number and size of nuclei in relation to the number of crystal-like clusters as the temperature decreases. Crystal-like clusters do not steadily accumulate with increased supercooling, although the number of crystal-like clusters is slightly larger at faster cooling rates than at slower cooling rates. This suggests a counter-balanced relationship between crystal-like clusters and icosahedral-like clusters in supercooled liquids. Crystal-like clusters with atom number less than four are unstable compared with icosahedral clusters, often dissolving into supercooled liquid^22^. It is also found that the two liquid systems with different cooling rates have different behaviors when the temperature approaches 775 K, i.e., some crystal-like clusters grow in the supercooled liquid and the crystallization takes place at faster cooling rate, whereas disordered structure remains in the supercooled liquid at slower cooling rate. This result suggests that in the moderate cooling rate region, the formation of stable crystal-like cluster depends on its size, which is an incident during structural fluctuation controlled by thermal history rather than cooling rates^22^. Figure 4 shows the average potential energy of atoms in crystal-like clusters at different temperatures for cooling rates of 12.6 × 10^11 ^K/s and 11.4 × 10^11 ^K/s. It is found that below about 750 K the average potential energies of atoms in crystal-like clusters using a cooling rate of 12.6 × 10^11 ^K/s are lower than those using a cooling rate of 11.4 × 10^11 ^K/s, which promotes the growth of crystal-like clusters and triggers crystallization during cooling in the case of 12.6 × 10^11 ^K/s cooling rate.

### Difference in glass transition between moderate cooling rates and high cooling rates

**Figure 5:**
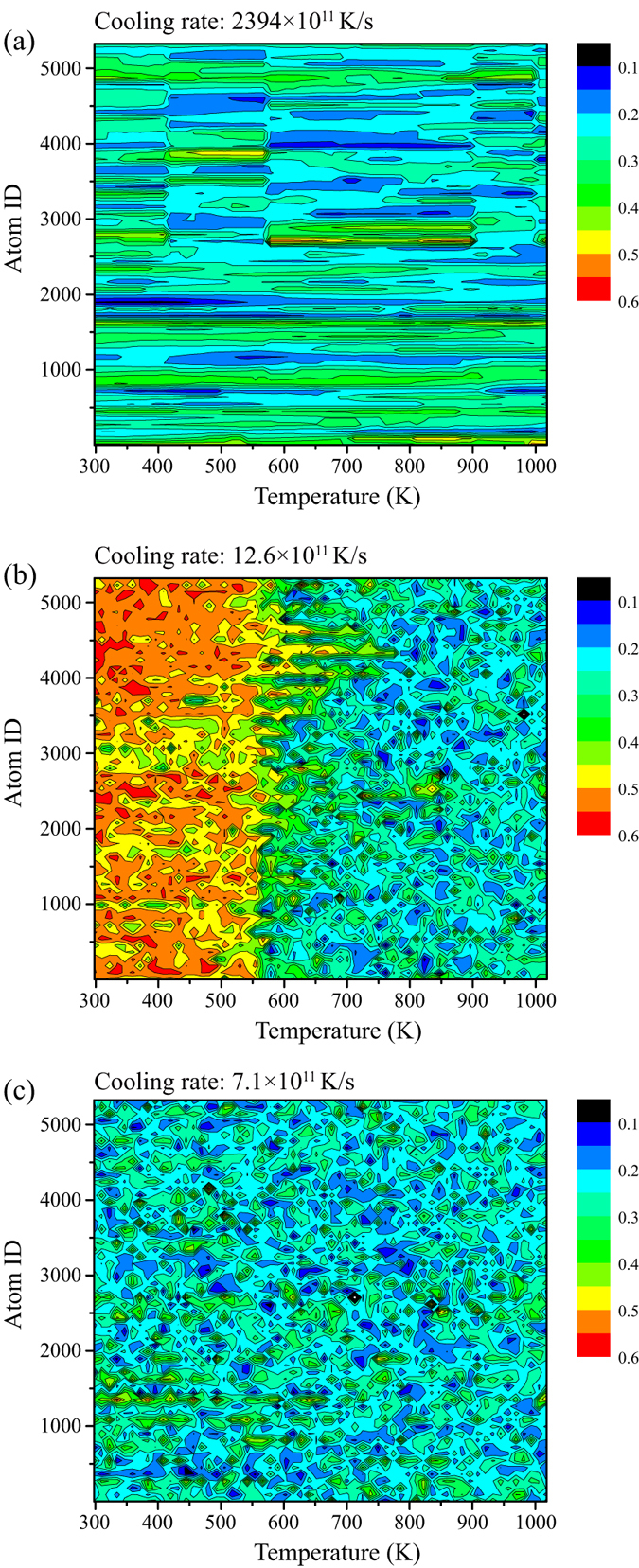
Evolution of local atomic structure. Contour maps of the occurrence of a configuration with values Q6 as a function of temperature for cooling rates of (**a**) 2394.0 × 10^11 ^K/s, (**b**) 12.6 × 10^11 ^K/s, and (**c**) 7.1 × 10^11 ^K/s, respectively.

We used BOO parameters to analyze the local structure evolution of supercooled liquid and study the difference in glass transition between a moderate cooling rate of 7.1 × 10^11 ^K/s and a high cooling rate of 2394.0 × 10^11 ^K/s. One case of crystallization produced by the cooling rate of 12.6 × 10^11 ^K/s is employed to clarify the mechanism of glass formation at a moderate cooling rate. The evolution of local atomic structure is identified by BOO parameters as introduced in the section of methods. Figure 5 shows the contour maps of the occurrence of a configuration with values *Q*_6_ as a function of temperature for cooling rates of (a) 2394.0 × 10^11 ^K/s, (b) 12.6 × 10^11 ^K/s, and (c) 7.1 × 10^11 ^K/s, respectively. These three cases respectively represent glass formation by a high cooling rate, crystallization and glass formation by moderate cooling rates. It is noted that the maximum points per dimension are set as 60 to facilitate visualization.

In the case of glass formation at a high cooling rate, the value of *Q*_6_ for each atom almost keep constant without significant change or fluctuation during the process of cooling, as seen in Fig. 5a. This indicates that the atomic structures featured by the symmetry of bond orientations in the supercooled liquid and glassy solid are similar to those in the liquid state. In other words, the final metallic glass in this case is produced by freezing the atomic structure of liquid to a large extent, without much structural relaxation and fluctuation. Also, the frozen is really compulsive although some slight crystal-like orders emerge during the process of cooling. However, things are quite different when the cooling rates reduce to the moderate region. As is shown in Fig. 5b,c, the values of *Q*_6_ vary approximately from 0.1 to 0.4, which locates in the typical region between icosahedral-like order and crystal-like order, at the early stage of supercooled liquid. These results indicate that the local atomic structures with icosahedral-like order and crystal-like order compete with each other constantly as the whole system searches for the minimum in the potential energy surface. The major reason for maintaining this type of competition is that the variations of bond orientations for the nearby atoms are highly non-resonant, which can be demonstrated by the dense contour lines, and the icosahedral-like order and crystal-like order transfer to each other since crystal-like clusters, if appear at some point, are unstable, owning to their small sizes (see the previous section). This structural fluctuation eventually results in the formation of metallic glass when the variations of bond orientations for the nearby atoms keep highly non-resonant in the whole process of cooling, as shown in Fig. 5c. Nevertheless, it is not always the case in the moderate cooling region. From Fig. 5b, as the temperature decreases to 750 K, values of *Q*_6_ for several atoms dramatically increase towards crystal-like order at a faster cooling rate of 12.6 × 10^11 ^K/s. Moreover, these crystal-orders seem have not tendency to turn back to the icosahedral-like order, but become more and more stable and also trigger the stabilization of crystal-like order for other surrounding atoms. As the number of atoms in the crystal-like cluster increases, the energy barrier of the crystalline-to-supercooled liquid reverse transformation process also increases^22^. Therefore, the phase transformation in the moderate cooling rate region depends on the structural fluctuation rather than cooling rates. Although it is not able to confirm the phase transformation at one given cooling process, the conclusion that a faster cooling rate cannot guarantee a lower crystal fraction near the critical cooling rate can be drawn. It is noteworthy that the “uncertainty” here is related to independent events rather than probability by statistics. The above results have manifested quite distinguishing behaviors of structure evolution when the liquids undergo high cooling rates and moderate cooling rates, we can then accordingly postulate two different mechanisms for glass formation: i) kinetic retardation of atom rearrangement and structural relaxation by high cooling rates and ii) competition of icosahedral order against crystal order at moderate cooling rates.

In conclusion, the abnormal phase transformations are frequently observed at cooling rates near the critical value for phase selection, resulting in the fluctuation of the crystalline fraction with cooling rates. Crystal nucleation occurs due to the emergence of adequate crystal-like clusters, while glass formation in this critical cooing rate region is determined by atomic structural fluctuation, controlled by thermodynamic factor, rather than kinetic factor. Two different mechanisms for glass formation are accordingly proposed. The results from this work extends our common understanding that glass formation is resulted from the kinetic freezing of supercooled liquid and also the common sense of cooling-rate dependent phase transformation.

## Methods

### MD simulations

MD simulations were performed on a cube with 5324 atoms subject to the three-dimensional periodic boundary conditions. In the simulation, we used a constant particle number, pressure, and temperature (NPT) ensemble; this method combines the Nosé canonical ensemble^23^ with variable shape and volume. NPT MD captures highly detailed microscopic information about a system, allowing for the study of phase transformation while also permitting the shape and volume of the system to change. The time interval was set at 1 fs to integrate Newton’s laws of motion. The MD simulations were carried out in temperatures increasing from 0 K to 1495 K, ten percent higher than the melting point of copper. After equilibrating the structure in the liquid state for 300 ps, we carried out two types of simulations, isothermal annealing and continuous cooling. The initial liquid structures were prepared by extending equilibrium times of 0 ps, 1 ps, and 10 ps, respectively, at 1495 K. To determine the TTT diagram for isothermal annealing, the liquid copper was momentarily quenched to temperatures ranging from 900 K to 500 K at intervals of 50 K. This range was selected for its proximity to the predicted nose temperature of 700 K, according to our observations during studies of phase transformation upon cooling. For the continuous cooling simulation, the system was continuously cooled to 298 K at cooling rates ranging from 4.0 × 10^11 ^K s^−1^ to 2394.0 × 10^11 ^K/s.

### BOO parameters

The local structure of particle *i* is characterized by the BOO parameters defined as follows^19^:


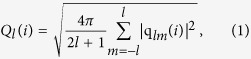


where


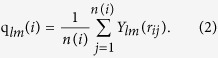


where, *n*(*i*) is the coordination number of particle *i. Y*_*lm*_(*r*_*ij*_) are spherical harmonics, where *r*_*ij*_ represents a vector from particle *i* to *j*, *l* is an integer, and *m* is an integer ranging from negative one to positive one. For metals, BOO analyses are commonly made at *l* = 4 and *l = *6^24^. In particular, *Q*_6_(*i*) is related to six-fold symmetry in three-dimensional space. Atoms with high values for *Q*_6_, greater than 0.2, serve as potential precursors for a crystal nucleus, while those with low *Q*_6_ less than 0.2 have a typical icosahedral-like order. In this work, we used *Q*_6_ to identify the tendency of crystal-like order and icosahedral-like order.

## Additional Information

**How to cite this article**: Han, J. J. *et al.* Abnormal correlation between phase transformation and cooling rate for pure metals. *Sci. Rep.* **6**, 22391; doi: 10.1038/srep22391 (2016).

## Supplementary Material

Supplementary Information
