# Supplementary material for: Abnormal correlation between phase transformation and cooling rate for pure metals
Source: Sci Rep. 2016 Mar 4;6:22391. doi: 10.1038/srep22391 (PMC4778031; doi:10.1038/srep22391)
Supplement: Supplementary Information [file srep22391-s1.pdf]

Supplementary Information

Abnormal correlation between phase transformation and cooling rate for pure metals

J. J. Han[1], C. P. Wang[1], X. J. Liu[1,2,*], Y. Wang[3], Z.-K. Liu[3], T.-Y. Zhang[4] and J. Z. Jiang[5]

1. Department of Materials Science and Engineering, College of Materials, Xiamen University, Xiamen 361005, P. R. China
2. Collaborative Innovation Center of Chemistry for Energy Materials, Xiamen University, Xiamen 361005, P. R. China
3. Department of Materials Science and Engineering, Pennsylvania State University, University Park, Pennsylvania 16802, U. S. A.
4. Shanghai University Materials Genome Institute and Shanghai Materials Genome Institute, Shanghai University, 99 Shangda Road, Shanghai 200444, China
5. International Center for New-Structured Materials (ICNSM), Laboratory of New-Structured Materials, State Key Laboratory of Silicon Materials, and School of Materials Science and Engineering, Zhejiang University, Hangzhou, 310027, P. R. China

*Corresponding author: lxj@xmu.edu.cn (X.J. Liu)

In this work, we have actually investigated more than a dozen pure metal systems (Cu, Ag, Au, Ni, Pd, Pt, Al, Pb, Fe, Mo, Ta, W, Ti, Zr). Figure S1 are the crystallinity as a function of cooling rates for iron with bcc structure and zirconium with hcp structure. It can be found that the phenomenon is universal.

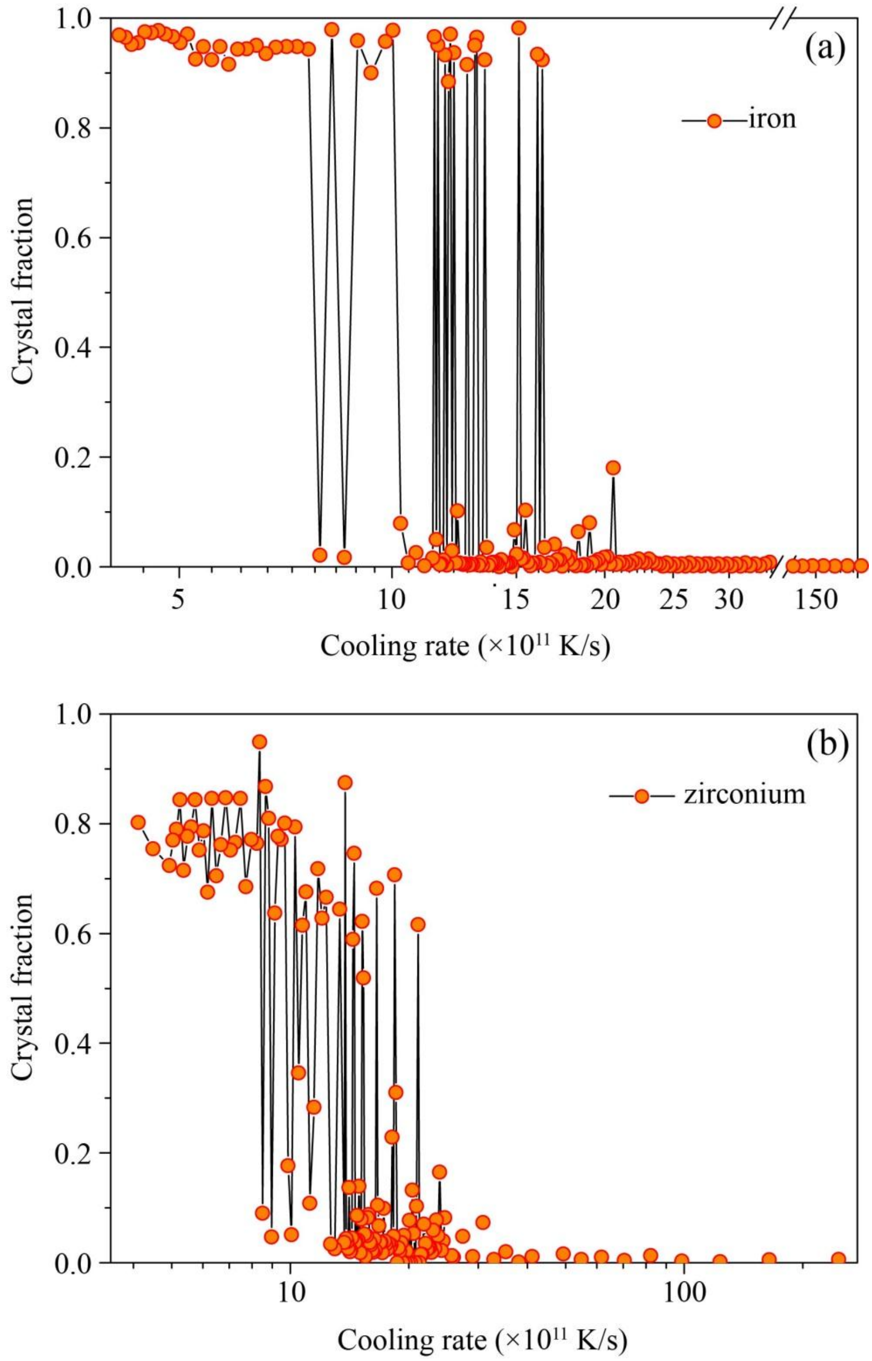

Figure S1. Crystallinity of **(a)** iron and **(b)** zirconium as a function of cooling rates.

We also examine the results using different system sizes of 11664 atoms and 49960 atoms. As shown in Fig. S2, the effect of system size on the results can be ignored. It should be noted that the crystal fraction of final crystal does not include the grain boundary, which results in the deviation of crystal fraction from 100% even though the supercooled liquid crystallizes totally.

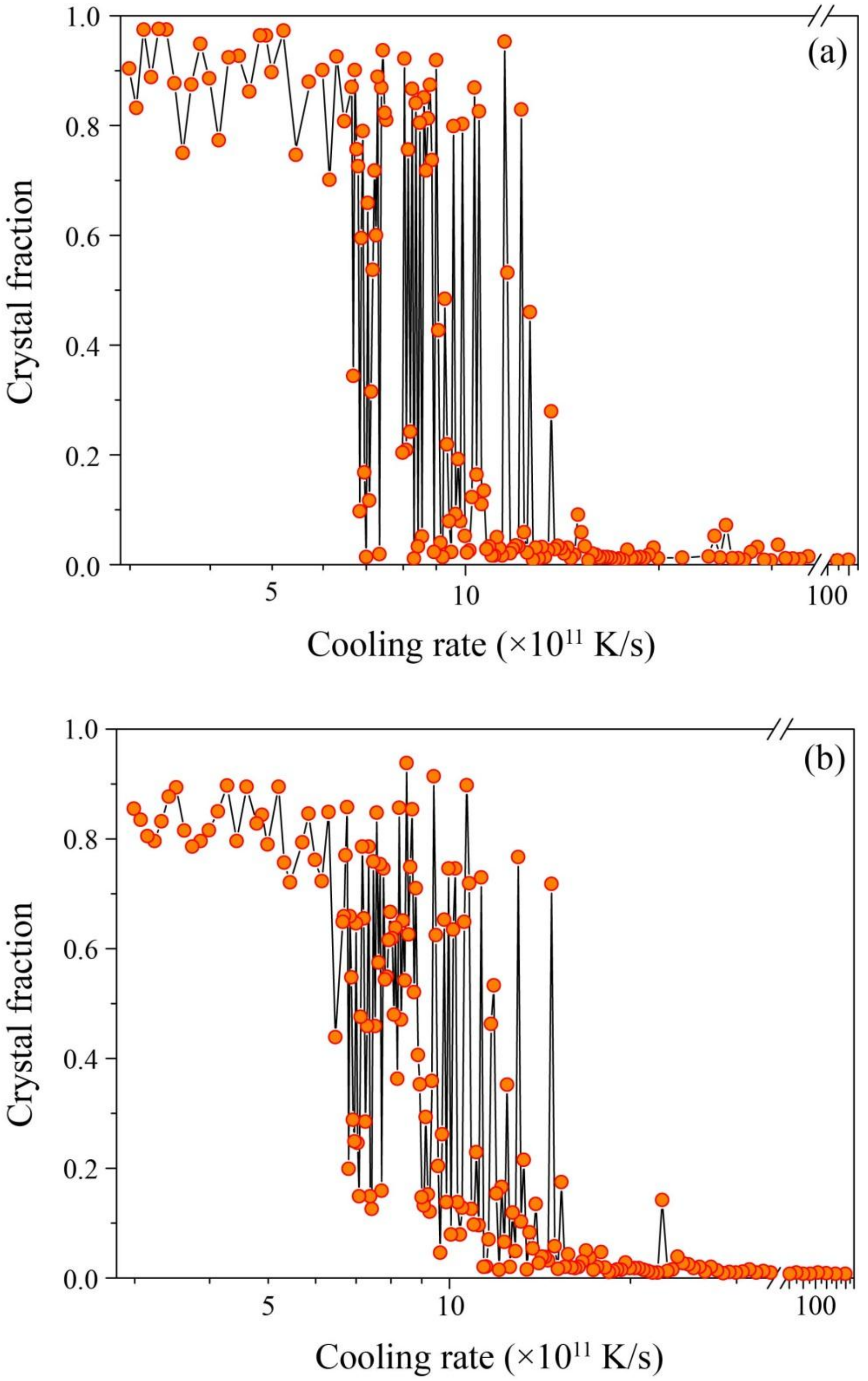

Figure S2. Crystallinity of copper as a function of cooling rates with **(a)** 11664 atoms and **(b)** 49960 atoms.
